# Comparative mapping of quantitative trait loci associated with waterlogging tolerance in barley (*Hordeum vulgare *L.)

**DOI:** 10.1186/1471-2164-9-401

**Published:** 2008-08-27

**Authors:** Haobing Li, René Vaillancourt, Neville Mendham, Meixue Zhou

**Affiliations:** 1 Tasmanian Institute of Agricultural Research and School of Agricultural Science, University of Tasmania, Private Bag 54, Hobart, TAS 7001, Australia; 2 School of Plant Science, University of Tasmania, Private Bag 55, Hobart, TAS 7001, Australia; 3 Tasmanian Institute of Agricultural Research and School of Agricultural Science, University of Tasmania, P.O. Box 46, Kings Meadows, TAS 7249, Australia

## Abstract

**Background:**

Resistance to soil waterlogging stress is an important plant breeding objective in high rainfall or poorly drained areas across many countries in the world. The present study was conducted to identify quantitative trait loci (QTLs) associated with waterlogging tolerance (e.g. leaf chlorosis, plant survival and biomass reduction) in barley and compare the QTLs identified across two seasons and in two different populations using a composite map constructed with SSRs, RFLP and Diversity Array Technology (DArT) markers.

**Results:**

Twenty QTLs for waterlogging tolerance related traits were found in the two barley double haploid (DH) populations. Several of these QTLs were validated through replication of experiments across seasons or by co-location across populations. Some of these QTLs affected multiple waterlogging tolerance related traits, for example, QTL Q_wt_4-1 contributed not only to reducing barley leaf chlorosis, but also increasing plant biomass under waterlogging stress, whereas other QTLs controlled both leaf chlorosis and plant survival.

**Conclusion:**

Improving waterlogging tolerance in barley is still at an early stage compared with other traits. QTLs identified in this study have made it possible to use marker assisted selection (MAS) in combination with traditional field selection to significantly enhance barley breeding for waterlogging tolerance. There may be some degree of homoeologous relationship between QTLs controlling barley waterlogging tolerance and that in other crops as discussed in this study.

## Background

Waterlogging is one of the major restrictions for barley production in high rainfall areas. It causes chlorophyll, protein and RNA degradation and also decreases the concentration of nutrients such as nitrogen, phosphorus, metal ions and minerals in barley shoots. These can occur rapidly after the onset of flooding, precede leaf chlorosis [1-3], and consequently reduce shoot and root growth, dry matter accumulation and final yield [4-8]. The average yield loss due to waterlogging is estimated to be 20–25% and can exceed 50% depending on the stage of plant development affected [9].

Barley cultivars differ in their tolerance to waterlogging. The barley collections from China, Japan and Korea contained many tolerant cultivars while those from North Africa, Ethiopia and southwest Asia showed few tolerant cultivars [10]. Fufa and Assefa [11] suggested that locally adapted landraces could be major sources of tolerance. Our previous studies showed some Chinese cultivars showed significantly better tolerance than Australian cultivars [12-14]. Thus it is possible to breed for tolerance. However, waterlogging tolerance is likely to be a complex trait affected by several mechanisms and complicated by confounding factors such as temperature, plant development stage, nutrient availability, soil type and sub-topography. Direct selection on grain yield has low effectiveness since the heritability of yield after waterlogging has been reported to be very low [15]. Different traits have been used as indirect selection indices for waterlogging tolerance. Among them, leaf chlorosis after waterlogging is one of the major indices used by researchers in different crops such as wheat (*Triticum spp*.) [16-19], soybean (*Glycine max*) [20]and barley [21]. Waterlogging tolerance has been found to be controlled by one dominant gene in common wheat [18], Makha wheat (*Triticum macha*) [22]and maize (*Zea mays ssp*. mays) [23]. In barley, based on leaf chlorosis, waterlogging tolerance was found to be a quantitative trait and mainly controlled by additive genetic variation [12,24]. Even though the heritability was relatively high for leaf chlorosis [12] and early generation selection could be efficient, well-controlled waterlogging conditions are still crucial for the precise evaluation of this trait. In practice, it is very difficult for breeders to control the multiple confounding environmental factors in a field experiment over thousands of barley genotypes. Development of molecular markers associated with barley waterlogging tolerance and marker assisted selection (MAS) could effectively avoid environmental effects. QTL analysis has proven to be very useful in identifying the genetic components of the variation for important economic traits [25]. A molecular marker closely linked to the target gene or QTL can act as a "tag" which can be used for indirect selection of the gene(s) in a breeding programme [26]. Great progress in molecular mapping of economically important traits in barley has been made [27]. Little progress, however, has been made in mapping QTLs controlling waterlogging tolerance in barley because it is affected by many factors in the natural environment [28]. With recent research showing that leaf chlorosis and some other physiological traits may be practical to use in the evaluation of waterlogging tolerance in barley [13,14], QTL identification has become possible. In this paper, we report on the identification of QTLs for waterlogging tolerance in two barley double haploid (DH) populations based on leaf chlorosis, plant survival and biomass reduction after waterlogging and comparisons were made between different populations and under different growing seasons.

## Methods

### Populations used for QTL analysis

The first population consisted of 92 doubled haploid (DH) lines from a cross between TX9425 and Franklin. TX9425 is a feed barley with waterlogging tolerance and originates from China, while Franklin is an Australian malting barley and is susceptible to waterlogging. The two parents also differ in malting quality, resistance to some diseases and several agronomic traits[29]. The second population consisted of 177 doubled haploid lines from the barley cross between Yerong and Franklin. Yerong is an Australian six-rowed variety with good tolerance to waterlogging stress.

### Map construction

#### DArT protocol

Genomic representations and preparation of the "discovery arrays" and "polymorphism-enriched arrays" were the same as explained by Wenzl et al. [30]. A quality parameter Q, which is the variance of the hybridization intensity between allelic states as a percentage of the total variance, was calculated for each marker. Only markers with a Q and call rate both greater than 80% were selected for linkage analysis.

#### SSR analysis

142 SSR primers were screened for polymorphism between the four parents of the two populations and 104 primers showed polymorphisms. Twenty-eight polymorphic primers were selected for genotyping the DH populations using four well-separated primers for each of the seven chromosomes.

#### AFLP analysis

AFLP markers were assayed only in the Franklin/TX9425 population. AFLP methodology was performed following Vos et al [31] with minor modification: Genomic DNA (250 ng) from the two parents and the DH lines was restricted with 2.5 u each of *Eco*RI and *Mse*I in a 20 μL reaction mixture for 2 hours at 37°C. Ligation mixtures of 20 μL containing the EcoRI and MseI adaptors, 1 U T4 DNA ligase, 0.4 mM ATP in 10 mM Tris-HCl (pH 7.5), 10 mM magnesium acetate, and 50 mM potassium acetate were added. Ligation mixtures were incubated at 16°C overnight. The reagents and thermo-cycling conditions for pre-selective and selective amplification followed Vos et al [31]. Pre-selective primers (EcoRI +A, MseI +C) and selective amplification primers (EcoRI +3, MseI +3) were described by Freeman et al [32]. The selective EcoRI (+3) primers were fluorescently labelled with TET for detection by a Gel Scan 2000. AFLP samples from the selective amplification were combined with two volumes of formamide B-blue loading buffer (98% v/v formamide, 10 mM EDTA, 0.25% w/v bromophenol blue, 0.25% w/v xylene cyanol) and denatured at 90°C for 3 min. Two μL of each sample was loaded onto 18 cm 6% w/v denaturing polyacrylamide gel with 7.0 M urea and electrophoresed in a 1% v/v TBE buffer at 1400 V for 1.5 h. Gene Profiler 4.03{3} software was used to extract data and score the traces. AFLP fragments were given a three-point confidence rating denoting their quality and ease of scoring. All AFLP markers were named using a code for each primer combination, followed by sequential numbers for scored bands e.g. p3b1.

#### Linkage analysis

The segregation signatures of each of the two individual datasets were imported into JoinMap 3.0 to distribute loci into linkage groups. LOD thresholds (from LOD 3 to LOD 10) were tested to group the markers, until a LOD threshold was obtained for each population that resulted in the optimum number of markers in linkage groups in which linkage order and distances were maintained. Marker order analyses were conducted with a JMMAP LOD threshold of 0.1 and a REC threshold value of 0.45. In order to obtain a rigorous marker order, framework maps were constructed using only non-distorted markers. Some distorted markers were then added into the data set gradually and integrated into the map frameworks. In most cases, the introduction of distorted markers did not affect the statistical confidence of marker order, or just changed the order of markers within small regions with high marker density. The genetic linkage map from the population of TX9425/Franklin comprised 412 DArT, 80 AFLP and 28 microsatellite markers and the map from the population of Yerong/Franklin comprised 496 DArT and 28 microsatellite markers.

### Evaluation of waterlogging tolerance of the DH lines

Four replicates of ten seeds for each DH and parental line were sown in soil in 3.5 L pots (one pot of each line per replicate) filled with soil from a frequently waterlogged site (Cressy Research Station) in Tasmania. Several measures were adopted to reduce the effects of variation in the degree of soil compaction across pots and also other sources of variation on the waterlogging conditions. First, the same type of pots was used through all the experiments. Second, we measured the same amount of soil for each pot and made sure the soil was packed to the same level in each pot. Third, the bottom of the water tanks or pools were checked to ensure they were flat and level. Finally, seeds were sown at the same depth in each pot.

After germination, five plants were kept in each pot and grown in a glasshouse under natural daylight but temperature controlled to less than 24°C. Waterlogging treatments were conducted in children's paddling pools. Each replicate was placed into a different pool and the two populations were placed in pools of different size. A randomised design was used for each pool. Three replicates were subjected to waterlogging and one replicate was not waterlogged as a control for the experiments. Waterlogging was achieved by filling the pool with water to just cover the soil surface in the pots. Waterlogging was started at the 3-leaf stage, and lasted three to eight weeks depending on the trait measured. This experiment was carried out in 2004 and repeated in 2005 using fresh soil and seeds.

The first trait measured was the proportion of each leaf that had lost its green colour (was yellow), this trait was called *leaf chlorosis*. Leaf chlorosis was chosen as the main indicator for waterlogging tolerance because other studies have found it to be correlated with yield reduction resulting from waterlogging stress [33]. This trait was measured three times for each population across the two experimental years (Table 1). Leaf chlorosis was measured as follows: the proportion of yellowing or chlorosis on each leaf was visually scored, then the length of each leaf was measured to weight the overall average proportion of chlorosis in each plant. Then an average was calculated for all the plants in each pot. The control plants of both populations in both years had no leaf chlorosis.

**Table 1:** Traits measured in the two barley mapping populations.

Traits measured in each population	Year of measurement		Duration of waterlogging stress
		
	2004	2005	
Franklin/TX9425			
Leaf chlorosis 1.1	×		two weeks
Leaf chlorosis 1.2	×		four weeks
Leaf chlorosis 2.1		×	two weeks
Plant survival	×		eight weeks
Plant biomass reduction		×	three weeks
Franklin/Yerong			
Leaf chlorosis 1.1	×		two weeks
Leaf chlorosis 2.1		×	two weeks
Leaf chlorosis 2.2		×	four weeks
Plant survival		×	eight weeks
Plant biomass reduction	×		three weeks

The second trait measured was *plant biomass reduction*. This trait was measured in 2004 for the Franklin/Yerong population and in 2005 for the Franklin/TX9425 population (Table 1). After three weeks of waterlogging treatments, barley plants were cut at ground level and dried at 60°C for four days in an electric oven. The average plant dry weight was measured for each replicate in both the control and in waterlogging treatments. Plant biomass reduction was calculated by subtracting the average dry weight of plants grown in waterlogging conditions from that in the control, then dividing by the average dry weight in the control. The third measured trait was *plant survival*. After eight weeks of waterlogging, dead plants in each pot were counted after the water was drained. Measurements were done in 2004 for Franklin/TX9425 and in 2005 for Franklin/Yerong (Table 1). Plant survival was calculated as the numbers of surviving plants divided by the initial number of plants in each pot.

### Statistical analysis

Statistical analysis was undertaken to detect significance of genetic effects for each trait in each population and also to calculate broad-sense heritability. For each experiment, the following mixed-effects model was used: *Y*_*ij *_= μ + *r*_*i *_+ *g*_*j *_+ *w*_*jj*. _Where: *Y*_*ij *_= observation on the *j*th genotype planted in the *i*th replication; μ = general mean; *r*_*i *_= effect due to *i*th replication; *g*_*j *_= effect due to the *j*th genotype; *w*_*ij *_= error or genotype by replication interaction, where genotype was random and replicate treated as a fixed effect in analysis conducted using PROC MIXED of SAS. As part of the model checking procedure, SAS PROC UNIVARIATE was used to verify that the residuals were normally distributed. Broad-sense heritabilities were calculated for each trait as the ratio of the genetic variation (genotype) divided by phenotypic variation (due to genotype and residual). In order to calculate least square means for each genotype by trait by population by experiment combinations, PROC GLM was used with the same model as above, except that genotype was treated as a fixed effect. The normality of each trait distribution was checked using SAS PROC UNIVARIATE for skewness and kurtosis.

Using the software package MapQTL5.0 [34], QTLs were first analysed by interval mapping (IM), followed by composite interval mapping (CIM). The closest marker at each putative QTL identified using interval mapping was selected as a cofactor and the selected markers were used as genetic background controls in the approximate multiple QTL model (MQM) of MapQTL5.0. Logarithm of the odds (LOD) threshold values applied to declare the presence of a QTL were estimated by performing the genome wide permutation tests implemented in MapQTL version 5.0 using at least 1000 permutations of the original data set for each trait, resulting in a 95% LOD threshold between 2.7 and 3.0. One or two LOD support intervals around each QTL were established, by taking the two positions, left and right of the peak, that had LOD values of one and two less than the maximum [34], after performing restricted MQM mapping which does not use markers close to the QTL. The percentage of variance explained by each QTL (R^2^) was obtained using restricted MQM mapping implemented with MapQTL5.0.

## Results

### Phenotypic and genetic variation among the DH lines of the two populations

Leaf chlorosis, plant survival and plant biomass reduction following waterlogging stress showed normal distributions for both populations with no significant skewness and kurtosis. Summary statistics for each trait are presented in Table 2 for both populations. Transgression beyond the parental values was observed for all traits including those for which parental values hardly differed. There was significant variation between DH lines (genetic variation) in each population for all the measured traits (Table 2). The effect of replication was not significant for traits measured early in the experiments, but was significant for most traits measured later. The broad sense heritabilities of the various traits ranged from 0.71 to 0.11 in the Franklin/TX9425 population and from 0.57 to 0.20 in the Franklin/Yerong population (Table 2). Biomass reduction was the ratio of the biomass of waterlogged plants divided by their control. Since the control consisted of only one replicate, due to limited glasshouse space, the results for biomass reduction need to be treated with caution.

**Table 2:** Descriptive statistics of the investigated waterlogging traits in the Franklin/TX9425 and Franklin/Yerong DH populations, with means for each parent, minimum/maximum/mean values of DH lines, standard deviation (SD) and probability (Prob Z) of significant variation among DH lines, and estimated broad-sense heritability (H^2^).

	Mean for parents		DH lines					
	
Traits	Franklin	Other parent	Min.	Max	Mean	SD	Prob Z	H^2^
Franklin/TX9425								
Leaf clorosis 1.1	0.10	0.34	0.04	0.40	0.19	0.08	< 0.0001	0.56
Leaf chlorosis 1.2	0.21	0.34	0.10	0.54	0.30	0.09	< 0.0003	0.11
Plant survival	0.93	0.74	0.00	1.00	0.55	0.28	< 0.0005	0.31
Leaf chlorosis 2.1	0.05	0.34	0.02	0.35	0.16	0.09	< 0.0001	0.71
Plant biomass reduction	0.37	0.51	0.18	0.71	0.43	0.11	0.0075	0.30
Franklin/Yerong								
Leaf chlorosis 1.1	0.13	0.19	0.04	0.27	0.14	0.05	< 0.0001	0.34
Plant biomass reduction	0.28	0.44	-0.05	1.05	0.39	0.19	< 0.0001	0.22
Leaf chlorosis 2.1	0.05	0.24	0.00	0.27	0.09	0.06	< 0.0001	0.20
Leaf chlorosis 2.2	0.28	0.38	0.15	0.65	0.34	0.08	< 0.0001	0.57
Plant survival	0.22	0.20	0.00	1.00	0.30	0.23	0.003	0.25

### Identification of QTLs associated with waterlogging tolerance in Franklin/TX9425

**Figure 1:**
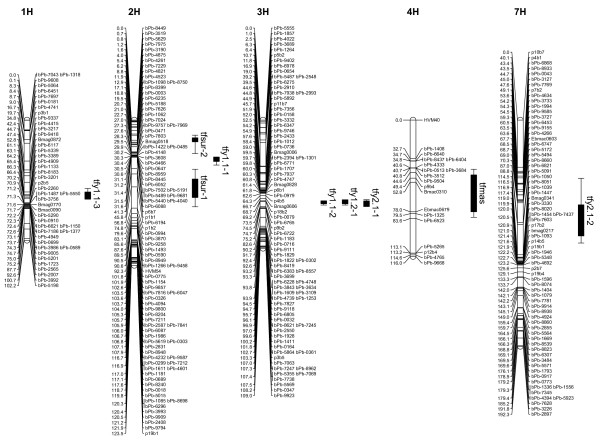
The Franklin/TX9425 chromosomes showing the locations of QTLs for the traits analyzed. Each linkage group consists of a vertical bar on which the map positions and names of loci are indicated. QTL positions are shown through their support interval on the right of each chromosome. One LOD support intervals are the inner intervals, while the outer intervals represent the two LOD support intervals. Prefix "bPb" and "p" signify a DArT marker and a AFLP marker, respectively. The other markers on the map are microsatellites.

Three QTLs (*tfy1.1-1, tfy1.1-2 *and *tfy1.1-3*) controlling leaf chlorosis after two-weeks of waterlogging stress (2004) were identified (Table 3, Figure 1). For all these QTLs, the Franklin alleles increased leaf chlorosis while the TX9425 alleles decreased it. One QTL (*tfy1.2-1*) was identified for leaf chlorosis after four-weeks waterlogging (2004) treatment. This is likely to be the same QTL as *tfy1.1-2 *as it was mapped to the same position and the Franklin allele also increased leaf chlorosis. Two QTLs (*tfy2.1-1 *and *tfy2.1-2*) were found for leaf chlorosis in the experiment carried out in 2005. QTL *tfy2.1-1 *is likely to be the same as *tfy1.1-2 *and *tfy1.2-1 *as it is in the same position and again the Franklin alleles increased leaf chlorosis.

**Table 3:** Characteristics of the detected QTLs explaining waterlogging related traits in the Franklin/TX9425 population.

Trait	QTL	Chr.	One LOD support interval (cM)	LOD score	*R*^2 ^(%)
Leaf chlorosis 1.1	*tfy1.1-1*	2H	31–35	9.21	23.3
(two weeks stress, 2004)	*tfy1.1-2*	3H	68–71	7.59	33.4
	*tfy1.1-3*	1H	61–67	2.75	7.1
					
Leaf chlorosis 1.2	*tfy1.2-1*	3H	67–71	7.31	36
(four weeks stress, 2004)					
					
Leaf chlorosis 2.1	*tfy2.1-1*	3H	68–73	9.28	34.1
(two weeks stress, 2005)	*tfy2.1-2*	7H	72–98	3.62	16
					
Plant biomass reduction	*tfmas*	4H	47–78	2.75	16.3
					
Plant survival	*tfsur-1*	2H	49–65	3.29	19
	*tfsur-2*	2H	15–18	2.75	13.2

Although the difference in the reduction of plant biomass due to waterlogging stress between TX9425 and Franklin was small (Table 2), one QTL (*tfmas*) was identified for plant dry weight reduction after three-weeks of waterlogging stress (Table 3). This QTL was mapped to chromosome 4H. Compared to the TX9425 allele, the Franklin allele led to a greater reduction of plant biomass following waterlogging.

Two QTLs (*tfsur-1 *and *tfsur-2*) were found for plant survival rate after eight weeks continuous waterlogging stress (Table 3). Both of these were located on chromosome 2H. These QTLs were located onto different regions of chromosome 2H compared with the QTLs for leaf chlorosis. This confirms the statistical analysis results showing no significant correlation between these two traits (results not shown). For the detected QTLs, the Franklin allele increased the survival rate of the plant at *tfsur-1 *locus, whereas TX9425 allele increased plant survival at the locus of *tfsur-2*. This may explain the strong transgressive segregation found for this trait.

### Identification of QTLs associated with waterlogging tolerance in Franklin/Yerong

**Figure 2:**
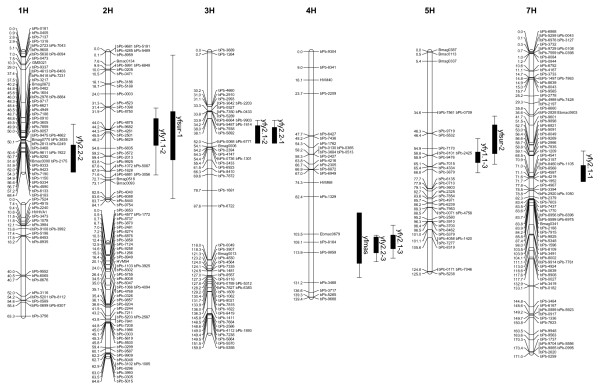
The Franklin/Yerong chromosomes showing the locations of QTLs for the traits analyzed. Each linkage group consists of a vertical bar on which the map positions and names of loci are indicated. QTL positions are shown through their support interval on the right of each chromosome. One LOD support intervals are the inner intervals, while the outer intervals represent the two LOD support intervals. Prefix "bPb" and "p" signify a DArT marker and a AFLP marker, respectively. The other markers on the map are microsatellites.

Two QTLs (*yfy1.1-1 *and *yfy1.1-2*) controlling leaf chlorosis after two-weeks of waterlogging stress (2004) were found on chromosome 2H and 5H. The Franklin alleles increased leaf chlorosis at the *yfy1.1-1 *locus, whereas at the *yfy1.1-2 *locus the Yerong allele increased leaf chlorosis (Table 4, Figure 2). Three QTLs (*yfy2.1-1, yfy2.1-2 *and *yfy2.1-3*) were found for leaf chlorosis after two weeks of waterlogging in the experiment carried out in 2005, these QTLs were located on chromosome 7H, 3H and 4H. The Franklin alleles increased leaf chlorosis in all three cases. Three QTLs (*yfy2.2-1, yfy2.2-2 *and *yfy2.2-3*) were found for leaf chlorosis after four weeks of waterlogging stress in the 2005 experiment, these QTLs were located on chromosome 3H, 1H and 4H. The Franklin allele increased leaf chlorosis at *yf2.2-1 *and *yf2.2-3 *loci, whereas the Yerong allele did so at the *yf2.2-2 *locus. QTL *yfy2.2-1 *is likely to be the same as *yfy2.1-2 *as it is in an identical position on chromosome 3H. The same applies to QTL *yfy2.1-1 *and *yfy2.2-3 *on chromosome 4H.

**Table 4:** Characteristics of the detected QTLs explaining waterlogging related traits in Franklin/Yerong population.

Trait	QTL	Linkage groups	One LOD support interval (cM)	LOD score	*R*^2 ^(%)
Leaf yellowing	*yfy1.1-1*	2H	46–55	2.90	5.8
proportion 1.1 (two	*yfy1.1-2*	5H	38–53	3.94	7.6
weeks stress, 2004)					
					
Leaf yellowing	*yfy2.1-1*	7H	64–73	3.72	6.7
proportion 2.1 (two	*yfy2.1-2*	3H	42–52	6.41	11.9
weeks stress, 2005)	*yfy2.1-3*	4H	104–112	9.25	18.5
					
Leaf yellowing	*yfy2.2-1*	3H	43–52	4.50	9.5
proportion 2.2 (four	*yfy2.2-2*	1H	53–68	2.77	5
weeks stress, 2005)	*yfy2.2-3*	4H	104–114	10.37	22.4
					
Reduction of plant biomass	*yfmas*	4H	91–120	3.03	8.2
					
Plant survival	*yfsur-1*	2H	34–61	3.15	7.1
	*yfsur-2*	5H	42–58	5.05	13.1

One QTL (*yfmas*) was identified for the reduction of plant biomass following waterlogging in this population (Table 4). This QTL mapped on chromosome 4H to almost the same position as QTL *yfy2.2-3 *and *yfy2.1-3 *and is probably due to pleiotropy. This was supported by the significant correlation between leaf chlorosis and plant biomass reduction in this population (results not shown).

Two QTLs (*yfsur-1 *and *yfsur-2*) were identified on chromosome 2H and 5H for plant survival rate after 8 weeks of continuous waterlogging stress. The Yerong allele increased plant survival rate at the *yfsur-1 *locus while the Franklin allele increased plant survival rate at the *yfsur-2 *locus. *Yfsur-1 *was located near *yfy1.1-1 *while *yfsur-2 *was located near *yfy1.1-2 *and again this may be because of pleiotropy.

### Comparison of waterlogging tolerance QTLs between populations

**Figure 3:**
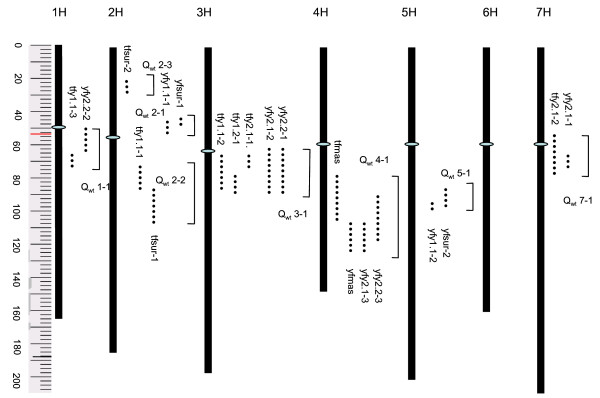
Comparison of quantitative trait loci (QTLs) identified for waterlogging tolerance in two different barley doubled haploid populations: tf = Franklin/TX9425; yf = Franklin/Yerong. Markers flanking the one LOD support interval of each QTL identified in the individual population were re-located on a barley composite map [35] so that their relative position could be compared. Centromeres are indicated as in [35]. A general name (such as Q_wt_1-1) was given to each chromosome region associated with waterlogging tolerance, the first number was the chromosome number and the second number was the serial number of regions identified on that chromosome.

In order to compare the QTLs identified in different populations, the markers flanking the one LOD support intervals for each QTL were relocated on the consensus map [35] including the two populations used in this study. Comparison of the identified QTLs between the two populations (Table 5; Figure 3) showed that many of the QTLs identified in Franklin/TX9425 mapped to similar chromosomal regions compared to those identified in Franklin/Yerong (such as QTLs identified on chromosome 3H and 7H), or mapped very close to one another with almost touching or overlapping two LOD support intervals (such as QTLs identified on chromosome 1 H, 2H and 4H) (Figure 3).

**Table 5:** Comparison of QTLs identified in the two populations after the flanking markers for each QTL were placed on the barley consensus map.

Chromosome	Franklin/Yerong			Franklin/TX9425		
	
	QTLs	Chromosome interval (cM)	Effect (%)	QTLs	Chromosome interval (cM)	Effect (%)
1H	*yfy2.2-2*	49–66	5	*tfy1.1-3*	68–73	7.1
						
2H	*yfy1.1-1*	47–56	5.8	*tfy1.1-1*	73–82	23.3
	*yfsur-1*	45–49	7.1	*tfsur-1*	82–115	19.1
				*tfsur-2*	26–33	13.2
						
3H	*yfy2.1-2*	59–90	11.9	*tfy1.1-2*	63–85	33.4
	*yfy2.2-1*	59–90	9.5	*tfy1.2-1*	78–97	36
				*tfy2.1-1*	63–68	34.1
						
4H	*yfy2.1-3*	114–136	18.6	*tfmas*	80–113	16.3
	*yfy2.2-3*	94–123	22.4			
	*yfmas*	114–136	8.2			
						
5H	*yfy1.1-2*	95–98	7.6			
	*yfsur-2*	86–98	13.2			
						
7H	*yfy2.1-1*	74–79	6.7	*tfy2.1-2*	50–83	16

## Discussion and conclusion

Leaf chlorosis in green plants is a complex and highly regulated process that occurs as part of plant development or that can be prematurely induced by stress. Recent analysis of the signalling pathways involved with different stress responses has indicated that these have considerable cross-talk with senescence related gene expression [3]. In wheat, many of the studies on waterlogging tolerance have been based on leaf chlorosis or leaf/plant death [18,36,37]. Leaf chlorosis has been found to be highly negatively correlated with grain yield which was regarded as the final criterion for waterlogging tolerance in wheat [33]. In barley, Hamachi et al [21] found that screening for waterlogging tolerance by the amount of dead leaf was a useful criterion and that the tolerance was under polygenic control, while Setter et al [9] concluded that severity of leaf chlorosis was not a good criterion. However, our preliminary yield trials using the same genetic material as used in our crosses (unpublished data) showed that under waterlogging conditions, the yield reductions of Franklin (which also has high leaf chlorosis under waterlogging) and TX9425 (low leaf chlorosis under waterlogging) were 86% and 28% in a pot experiment and 61% and 39% in a controlled field experiment (data not shown). Since leaf chlorosis after waterlogging showed high heritability [12], this trait was used as the major criterion to test for waterlogging tolerance along with plant survival, and plant biomass reduction in the current study.

The QTL analysis of two doubled haploid populations (Figure 3) found at least seven distinct QTLs for waterlogging tolerance. It was also demonstrated that some QTLs controlling leaf chlorosis were very stable and were validated under different stress duration, between different experiments and different populations (for example QTLs on chromosomes 1H, 3H and 7H). Some QTLs affected multiple waterlogging tolerance related traits, for example, the allele on chromosome 4H from the tolerant parent contributed not only to reducing barley leaf chlorosis, but also to increasing plant biomass under waterlogging stress, whereas other allelles such as those on chromosomes 2H and 5H controlled both leaf chlorosis and plant survival. This result suggested that leaf chlorosis is an important stable selection criterion for barley waterlogging tolerance, which can be used practically in breeding programs.

Waterlogging tolerance is a complex trait affected by several mechanisms and complicated by confounding factors such as temperature, plant development stage, nutrient, soil type and sub-topography. The current experiment was conducted under well controlled environmental conditions. The soil, obtained from a waterlogged site in Tasmania, was well mixed before being evenly packed into pots. Waterlogging treatments were conducted in the early vegetative growth stage to avoid the effect of variation in development rate on waterlogging tolerance. As indicated in the Material and Methods, the parents of both populations differ in many developmental traits including ear emergence in both populations and plant height in the Franklin/TX9425 population. One major QTL located on chromosome 2H was found for plant height and ear emergence in the Franklin/TX9425 population and two major QTLs located on chromosomes 2H and 7H were found for ear emergence in the Franklin/Yerong population (data not shown). The locus controlling row type in the Franklin/Yerong population was located on chromosome 2H, which is in a similar position to that reported in other studies [38]. None of these loci were within the confidence intervals of the QTLs controlling waterlogging tolerance detected in the current study.

Accuracy of QTL mapping is important in implementing marker-assisted selection (MAS) for polygenic traits, but small confidence intervals for QTL positions are not easily obtained [39,40], although typical approximate 95% confidence intervals for QTL positions are of the order of 20 cM [41,42]. Van Ooijen [40] recommended using a two LOD support interval as an approximation of the 95% confidence intervals. Using only the one LOD support interval in this study, we observed significant overlap in QTL positions across populations. The results of this study showed that one LOD support intervals around QTLs identified in the Franklin/Yerong population were smaller than those in the Franklin/TX9425 population, this is because the Franklin/Yerong population was larger and further reduction in size of confidence intervals will require the use of larger populations [43].

There is only one published report of QTLs for waterlogging tolerance in barley. Qian et al [44] found one SSR marker (WMC1E8) correlated with waterlogging tolerance based on chlorophyll content of the second top leaf in an F_2 _population by constructing two DNA (tolerant and susceptible) bulks. The identified QTL explained 29.9% of the total variation [44], and the authors deduced that this QTL was located on chromosome 1H based on the published barley linkage maps [45]. In our study we identified QTLs controlling leaf chlorosis in both populations on chromosome 1H. However, the position of the QTLs found in our study were different from that of WMC1E8 reported by Qian et al [44] according to the consensus map [35].

Different segregating populations of rice, maize, wheat, and barnyard grass have been studied for diverse waterlogging related characteristics or criteria, such as plant survival, leaf senescence, the extent of stimulation of shoot elongation caused by stress [46], waterlogged shoot growth and waterlogged root growth [47], adventitious root formation and leaf injury [48,49]. QTLs controlling many of these traits have been identified. Comparison of genetic mechanisms of waterlogging or flooding tolerance among different crops remains difficult because different waterlogging related traits were used for QTL analysis in these studies. Another difficulty for comparing QTLs identified for waterlogging tolerance in different species is the lack of common markers among different genetic linkage maps, sometimes even among different populations within the same species. Different marker nomenclature among researchers also contributes to the difficulties with comparative mapping.

Despite these difficulties, comparative mapping across cereals can provide interesting information. For example, a major QTL controlling waterlogging tolerance based on dry matter production in maize was located on chromosome 1 [50]. In our experiment, a QTL controlling plant biomass under waterlogging stress was identified on chromosome 4H, which comparative mapping has shown to be highly homoeologous to chromosome 1 in maize [51,52]. QTLs controlling percent plant survival in rice under submergence stress were mapped to chromosome 7, 9 and 10, and the QTL located on chromosome 9 was the most significant one [46]. According to comparative mapping in the grass family, rice chromosome 9 had a homoeologous relationship with wheat chromosome 5L and maize chromosome 2 [51]. Maize chromosome 2 is in part homoeologous to wheat chromosome 2 [51], so it can be deduced that rice chromosome 9 is homoeologous with barley chromosome 2H and 5H [52]. In barley, the QTLs contributing to plant survival were located on chromosomes 2H and 5H. These QTLs identified for controlling plant survival could be the same as the QTL identified on chromosome 7 and 9 in rice.

Improving waterlogging tolerance in barley is at an early stage compared with other traits. The future use of marker assisted selection (MAS) in combination with traditional field selection could significantly enhance barley breeding for waterlogging tolerance. As demonstrated in this study, and in other previously published studies [53], diversity array technology (DArT) is very efficient for whole-genome profiling [30]. Although this technique is still limited to only a few laboratories at this stage, barley consensus maps [35] have been constructed to link DArT markers with many SSR and RFLP markers which have been previously developed and applied widely in barley mapping studies and to provide plant breeders with practically useful molecular markers for improving barley waterlogging tolerance. DArT markers can easily be sequenced and to obtain stronger support for the microsynteny of the QTLs (or genes) for waterlogging tolerance among grass species, further research should involve direct comparison of DNA sequence of markers (those linked to QTLs) to that of the genome sequence of rice [54,55] and other species.

## Authors' contributions

HBL selected barley genotypes and made crosses between them for DH population construction, performed SSR and AFLP assays, prepared DNA samples for DArT assays, built the component maps and the composite map, screened waterlogging tolerance on the DH lines, conducted statistical analysis and QTL analysis, drafted the manuscript, figures and tables. REV supervised this project and provided technical guidance. NM supervised this project. MXZ constructed DH populations for Franklin/TX9425 and Franklin/Yerong crosses and supervised this project.
